# Mfa4, an Accessory Protein of Mfa1 Fimbriae, Modulates Fimbrial Biogenesis, Cell Auto-Aggregation, and Biofilm Formation in *Porphyromonas gingivalis*

**DOI:** 10.1371/journal.pone.0139454

**Published:** 2015-10-05

**Authors:** Ryota Ikai, Yoshiaki Hasegawa, Masashi Izumigawa, Keiji Nagano, Yasuo Yoshida, Noriyuki Kitai, Richard J. Lamont, Fuminobu Yoshimura, Yukitaka Murakami

**Affiliations:** 1 Department of Oral Microbiology, Asahi University School of Dentistry, Mizuho, Gifu, Japan; 2 Department of Orthodontics, Asahi University School of Dentistry, Mizuho, Gifu, Japan; 3 Department of Microbiology, School of Dentistry, Aichi Gakuin University, Nagoya, Aichi, Japan; 4 Department of Oral Immunology and Infectious Diseases, School of Dentistry, University of Louisville, Louisville, KY, United States of America; University of Illinois at Urbana-Champaign, UNITED STATES

## Abstract

*Porphyromonas gingivalis*, a gram-negative obligate anaerobic bacterium, is considered to be a key pathogen in periodontal disease. The bacterium expresses Mfa1 fimbriae, which are composed of polymers of Mfa1. The minor accessory components Mfa3, Mfa4, and Mfa5 are incorporated into these fimbriae. In this study, we characterized Mfa4 using genetically modified strains. Deficiency in the *mfa4* gene decreased, but did not eliminate, expression of Mfa1 fimbriae. However, Mfa3 and Mfa5 were not incorporated because of defects in posttranslational processing and leakage into the culture supernatant, respectively. Furthermore, the *mfa4*-deficient mutant had an increased tendency to auto-aggregate and form biofilms, reminiscent of a mutant completely lacking Mfa1. Notably, complementation of *mfa4* restored expression of structurally intact and functional Mfa1 fimbriae. Taken together, these results indicate that the accessory proteins Mfa3, Mfa4, and Mfa5 are necessary for assembly of Mfa1 fimbriae and regulation of auto-aggregation and biofilm formation of *P*. *gingivalis*. In addition, we found that Mfa3 and Mfa4 are processed to maturity by the same RgpA/B protease that processes Mfa1 subunits prior to polymerization.

## Introduction

*Porphyromonas gingivalis*, a gram-negative obligate anaerobic bacterium, is a component of dental plaque biofilms in humans and plays a key role in the initiation and progression of chronic periodontitis [1]. It is also thought to be associated with systemic diseases such as cardiovascular disease [2], rheumatoid arthritis [3], and nonalcoholic fatty liver disease [4]. The organism expresses a number of potential virulence factors, including fimbriae and gingipain proteases [5]. The type strain of *P*. *gingivalis*, ATCC 33277, expresses two forms of fimbriae, FimA and Mfa1, which are composed mostly of polymers of the corresponding proteins [6], and facilitate binding to host cells, matrix proteins, and other bacteria. On the other hand, gingipains are a family of proteolytic enzymes that comprises arginine-specific (RgpA and RgpB) and lysine-specific (Kgp) proteases, and participates in a wide range of pathological and physiological processes [7].

**Fig 1:**
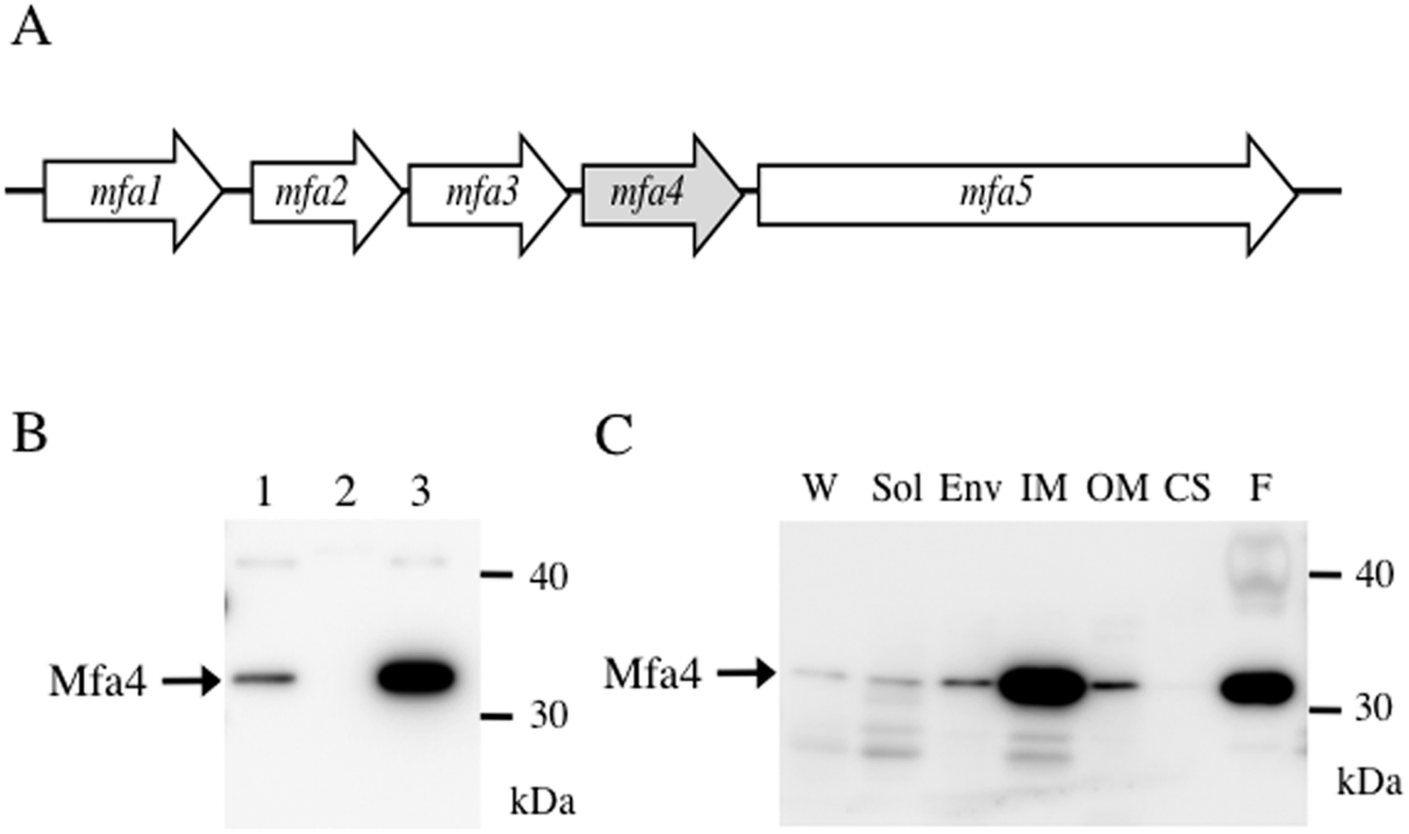
The *mfa4* gene product in *P*. *gingivalis*. (A) Schematic diagram of the *mfa* gene cluster in *P*. *gingivalis* ATCC 33277, based on sequence NC_010729 deposited in NCBI. The cluster contains five genes, *mfa1*-*5*, in the same transcriptional orientation. (B) Mfa4 expression. Whole-cell lysates from *P*. *gingivalis* JI-1 (lane 1), FMFA4 (lane 2), and FMFA4C (lane 3) were separated by SDS-PAGE, immunoblotted, and probed with Mfa4 antiserum. (C) Subcellular localization. A culture of *P*. *gingivalis* JI-1 was harvested by centrifugation. The culture supernatant (CS) was collected and proteins were concentrated by ammonium sulfate precipitation. Whole-cell lysates (W), obtained by lysing cells through French press, were fractionated into soluble (Sol), envelope (Env), inner membrane (IM), and outer membrane (OM) fractions. Mfa1 fimbriae (F) were purified from the soluble fraction and used as positive control. Samples were separated by SDS-PAGE and subjected to immunoblotting using anti-Mfa4 as probe.

Mfa1 fimbriae have been examined independently by two groups [8, 9]. The Mfa1 fimbria is 0.1–0.5 μm in length, while the FimA fimbria is over 1 μm [10]. Subsequent analysis of purified Mfa1 fimbriae indicates that they are helical structures with pitch ca. 27 nm and average length 103 nm [11]. The *mfa1* gene is in a gene cluster consisting of five genes, namely *mfa1*, *mfa2*, *mfa3*, *mfa4* (formally PGN_0290), and *mfa5* (PGN_0291) (Fig 1A). The *mfa* cluster is typically transcribed as a four-gene polycistronic message encompassing *mfa1-mfa4* [12].

We have characterized gene products from this cluster in terms of structure and function in Mfa1 fimbriae. We previously demonstrated that Mfa2 anchors Mfa1 fimbriae to the outer membrane and regulates fimbrial length [12]. In addition, we have shown that Mfa3, Mfa4, and Mfa5 co-purify with Mfa1 fimbriae and are therefore considered accessory proteins [12]. Furthermore, we found that Mfa3 caps the distal tip of Mfa1 filaments [13]. Recent studies indicate that Mfa1 fimbriae suppress and regulate homotypic biofilm development in *P*. *gingivalis* [14]. It was also reported that Mfa1 fimbriae are essential for coadhesion and community formation with oral commensal bacteria such as *Streptococcus gordonii* [11] and for survival within human myeloid dendritic cells [15]. Indeed, we recently reported that an *mfa3* mutant, which lacks all accessory proteins in the fimbriae, strongly auto-aggregates and forms biofilms, indicating that accessory proteins restrict auto-aggregation and biofilm formation [13].

Mfa3, with molecular weight 43 kDa, temporarily accumulates in the inner membrane and is then proteolytically processed. The 40-kDa mature Mfa3 redistributes to the outer membrane, where it is finally incorporated into fimbriae [13]. N-terminal sequencing suggests that Mfa3 is processed by gingipains. Therefore, it has been proposed that this accessory protein is processed and transported from the cytoplasm to the outer membrane in the same manner as the major structural proteins FimA and Mfa1 [16, 17], which are also processed by the gingipain RgpA/B to yield mature forms [18, 19].

In this study, we characterize Mfa4 and its role in the biogenesis and assembly of functional Mfa1 fimbriae. Furthermore, we show that RgpA/B is involved in the maturation of the accessory proteins Mfa3 and Mfa4.

## Materials and Methods

### Bacterial strains, plasmids, and culture

Bacterial strains and plasmids are summarized in Table 1. *P*. *gingivalis* was cultivated anaerobically at 37°C on Brucella HK agar (Kyokuto Pharmaceutical Industrial, Tokyo, Japan) supplemented with 5% (v/v) laked rabbit blood, 2.5 μg/mL hemin, 5 μg/mL menadione, and 0.1 μg/mL dithiothreitol (DTT). Liquid cultures were grown in trypticase soy broth supplemented with 0.25% (w/v) yeast extract, 2.5 μg/mL hemin, 5 μg/mL menadione, and 0.1 μg/mL DTT (sTSB). Where appropriate, media were supplemented with 5 μg/mL chloramphenicol, 20 μg/mL erythromycin, or 1 μg/mL tetracycline. *Escherichia coli* was grown in Luria-Bertani media supplemented as needed with 50 μg/mL ampicillin, 50 μg/mL kanamycin, or 200 μg/mL erythromycin.

**Table 1:** Bacterial strains and plasmids.

Strain or plasmid	Genotype and relevant description^1^	Source
***Porphyromonas gingivalis***		
ATCC 33277	Type strain, Gm^r^	ATCC^2^
JI-1	*fimA* deletion mutant from ATCC 33277, Cm^r^	[12]
JI-12	*mfa2*-deficient mutant from JI-1, Cm^r^ Em^r^	[12]
FMFA4	*mfa4*-deficient mutant from JI-1, Cm^r^ Em^r^	This study
FMFA4C	FMFA4 complemented with *mfa4* through pTCOW::*ragAP*::*mfa4*, Cm^r^ Em^r^ Tc^r^	This study
KDP98	*fimA*-deficient mutant from ATCC 33277, Em^r^	[20]
KDP112	*rgpA/B*-deficient mutant from ATCC 33277, Tc^r^ Em^r^	[21]
SMF-fimA	*fimA* and *mfa1* double mutant from ATCC 33277, Cm^r^ Em^r^	[13]
FMFA3	*mfa3* deletion mutant from KDP98, Cm^r^ Em^r^	[13]
FMFA5	*mfa5*-deficient mutant from JI-1, Cm^r^ Em^r^	This study
***Escherichia coli***		
TOP10	F- *mcr*A Δ(*mrr*-*hsd*RMS-*mcr*BC) φ80*lac*ZΔM15 Δ*lac*Χ74 *rec*A1 *ara*D139 Δ(*ara*-*leu*) 7697 *gal*U *gal*K *rps*L (Str^R^) *end*A1 *nup*G λ-	Thermo Fisher Scientific Inc.
S17-1	Used to deliver pT-COW to *Bacteroides* and *P*. *gingivalis* via conjugation	[22]
**Plasmids**		
pVA2198	Source of the drug cassette *ermF-ermAM*, Em^r^	[23]
pCR-Blunt II-TOPO	Cloning vector, Km^r^	Invitrogen
pTCOW::*ragAP*	pTCOW derivative containing a *ragA* promoter; Ap^r^ in *E*. *coli*, Tc^r^ in *P*. *gingivalis*	[24]
pTCOW::*ragAP*::*mfa4*	pTCOW::*ragAP* derivative containing *mfa4* ORF; Ap^r^ in *E*. *coli*, Tc^r^ in *P*. *gingivalis*	This study

^1^ Ap^r^, ampicillin resistance; Cm^r^, chloramphenicol resistance; Em^r^, erythromycin resistance; Gm^r^, gentamicin resistance; Km^r^, kanamycin resistance; Tc^r^, tetracycline resistance; Str^r^, streptomycin resistance.

^2^ ATCC, American Type Culture Collection.

### DNA manipulations

Restriction endonucleases and DNA ligase were purchased from Takara Bio Inc. (Otsu, Japan) or New England Biolabs (Ipswich, MA, USA). PCR primers (Table 2) were synthesized by Sigma Genosys (Ishikari, Japan) and were used in standard PCR using PCR Thermal Cycler Dice™ (Takara Bio Inc.) and Pyrobest (Takara Bio Inc.), a high-fidelity DNA polymerase.

**Table 2:** Primers.

Primer	Sequence (5’-3’)
Mfa4F	GGCGAGTAGGTACCACGAACCAATGGCTTGG
Mfa4R	TGAATTAAGGTACCCTTATTTGCAGGCACC
cMfa4F	AATAGACTCTAGAATGAAAAAGTATTTGTTATATG
cMfa4R	AAGACCTGCGGCCGCTCAAATCTCGACTTCGTACTTGT
Mfa5F	TCTAAATTGGTACCTGCAAATAAGGGTAAC
Mfa5R	TTGACAGGGGTACCGAAATACTGCCAGCTC

Restriction sites are underlined.

### Construction of gene-deficient and complemented strains

To construct the *mfa4*-deficient mutant, an internal fragment of the *mfa4* gene was amplified by PCR with primers Mfa4F and Mfa4R (Table 2), the sequences of which were designed based on the *P*. *gingivalis* ATCC 33277 genome (NC_010729) [25]. The amplified 2.0-kbp fragment was ligated into a pCR-Blunt II-TOPO plasmid vector (Thermo Fisher Scientific Inc. Carlsbad, CA) and propagated in *E*. *coli* TOP10 (Thermo Fisher Scientific Inc.). The *mfa4* gene was disrupted by inserting into the *Bal*I site a fragment containing *ermF*, which is expressed in *Porphyromonas* and *Bacteroides* spp., and *ermAM*, which is expressed in *E*. *coli*. This fragment was excised from plasmid pVA2198 [23] by digestion with *Kpn*I and *Bam*HI.

The plasmid containing disrupted *mfa4* was linearized with *Kpn*I, and then delivered by electroporation into *P*. *gingivalis* JI-1, a *fimA*-deficient strain (Table 1). After 12 h of anaerobic incubation in sTSB, cells were spread on Brucella HK agar supplemented with 20 μg/mL erythromycin and grown anaerobically at 37°C for 7 days.

The *mfa5* disruption mutant was constructed in a similar manner. In brief, an internal sequence of *mfa5* was amplified by PCR with primers Mfa5F and Mfa5R (Table 2), ligated into pCR-BluntII-TOPO, and amplified in *E*. *coli* TOP10. The erythromycin-resistance cassette as described was inserted into the *Sac*I site in the *mfa5* fragment. The resulting plasmid was linearized and then delivered by electroporation into *P*. *gingivalis* JI-1. Specific gene disruptions in *mfa4* and *mfa5* were confirmed by PCR and Southern blotting (data not shown).

To obtain the complemented strain FMFA4C, *mfa4* was amplified by PCR from ATCC 33277 using cMfa4F and cMfa4R (Table 2) and inserted downstream of the *ragA* promoter in pTCOW::*ragAP* (Table 1), which is derived from pT-COW [24, 26]. The resulting vector, pTCOW::*ragAP*::*mfa4*, was delivered into FMFA4 via conjugation from *E*. *coli* S17-1 [22].

### Purification of Mfa1 fimbriae

Mfa1 fimbriae were purified from *P*. *gingivalis* JI-1, FMFA4, and FMFA5 as described previously [13]. Briefly, cells were lysed by French press. The soluble fraction was cleared by ultracentrifugation and precipitated with ammonium sulfate at 50% saturation. Mfa1 fimbriae were purified by ion exchange and size exclusion chromatography. Purity and identity were verified by SDS-PAGE and mass spectrometry.

### Subcellular fractionation

*P*. *gingivalis* strains were cultivated in sTSB until the early stationary phase. The culture supernatant was separated from bacterial cells by centrifugation and proteins were concentrated by ammonium sulfate precipitation at 70% saturation, as described previously [27]. Cells were resuspended in 10 mM HEPES-NaOH pH 7.4 containing 0.1 mM N-α-p-tosyl-L-lysine chloromethyl ketone, 0.2 mM phenylmethylsulfonyl fluoride, and 0.1 mM leupeptin, and then lysed by French press. Residual intact cells were removed by centrifugation at 1,000 ×*g* for 10 min. The whole-cell lysate was then fractionated as previously described [28]. Briefly, the soluble fraction was separated from the envelope fraction by centrifugation at 100,000 ×*g* for 60 min at 4°C. The envelope fraction was suspended for 30 min at 20°C in HEPES buffer pH 7.4 containing 1% Triton X-100 and 20 mM MgCl_2_ and then centrifuged at 100,000 ×*g* for 60 min at 4°C. The resulting supernatant contained the inner membrane fraction, while the sediment contained the outer membrane fraction and was subsequently homogenized in HEPES buffer.

### SDS-PAGE and immunoblotting

Samples containing 5 μg total protein were solubilized in a buffer containing SDS and 2-mercaptoethanol and then heated at 100°C for 5 min, 80°C for 10 min, or 60°C for 10 min. Subsequently, samples were separated using SDS-PAGE, and were either stained with Coomassie brilliant blue R-250 or blotted on PVDF membranes. Membranes were blocked with 5% skim milk in 20 mM Tris-HCl pH 7.4 with 300 mM NaCl and 0.05% Tween 20. Membranes were then probed with primary rabbit polyclonal antibodies against purified Mfa1 fimbriae, Mfa2, Mfa3, Mfa4, or Mfa5 [12, 13], and labeled with secondary HRP-conjugated goat anti-rabbit IgG (MP Biomedicals, Santa Ana, CA). Finally, bands were visualized with ECL-prime (GE Healthcare, Buckinghamshire, UK).

### N-terminal sequencing

Proteins separated by SDS-PAGE were electrophoretically transferred to PVDF membranes and stained with Coomassie brilliant blue R-250. Mfa4 bands were excised and analyzed by N-terminal sequencing on an ABI 477 A automatic peptide sequence analyzer at Center for Instrumental Analysis, Hokkaido University.

### Mass spectrometry

After SDS-PAGE, proteins of interest were analyzed using matrix-assisted laser desorption ionization time-of-flight mass spectrometry (MALDI-TOF MS) as described previously [29]. Briefly, bands were digested with trypsin in-gel, and digestion products were extracted, concentrated, and analyzed using a 4800 MALDI TOF/TOF Analyzer (AB Sciex, Framingham, MA). Proteins were identified from MS peaks using MS-Fit peptide mass fingerprinting methods in the Mascot program (http://www.matrixscience.com/).

### Preparation of antiserum against Mfa1-only fimbriae

Antiserum against Mfa1 fimbriae consisting of Mfa1 only and without accessory proteins was prepared according to published methods [13]. Briefly, Mfa1 fimbriae that lack all accessory proteins (Hasegawa, unpublished data) were purified from strain FMFA5, mixed with Freund’s complete adjuvant, and subcutaneously injected into rabbits three times at 2-week intervals. A rabbit was housed (36 cm x 35 cm x 48 cm) in the climate-controlled room (23.3 ± 0.2°C, relative humidity of 58 ± 9%) maintained on a light/dark cycle with lights on from 07:00 to 19:00 with free access to food and water. During procedures, health monitoring was conducted every day. Animal work was performed in strict accordance with Regulations on Animal Experimentation at Aichi Gakuin University including humane endpoints. Protocols were approved by the Aichi Gakuin University Animal Research Committee (Permit No. AGUD113). All efforts were made to minimize animal suffering, and animals were sacrificed under sodium pentobarbital anesthesia.

### Filtration ELISA

To detect Mfa1 fimbriae exposed on the cell surface, filtration ELISA was performed using intact cells as antigen [30]. A 100 μL cell suspension containing 5×10^6^ cells was applied over filters in a 96-well MultiScreen-GV filtration plate with pore size 0.22 μm (EMD Millipore, MA). Subsequently, wells were washed with a buffer consisting of 20 mM Tris pH 7.5, 150 mM NaCl, and 0.05% Tween 20 (TBST), and blocked with 3% bovine serum albumin in TBST. Wells were then probed with a 1,000-fold dilution of antiserum against Mfa1-only fimbriae, washed, and then labeled with HRP-conjugated polyclonal goat anti-rabbit IgG (Dako, Glostrup, Denmark). Subsequently, *o*-phenylenediamine and H_2_O_2_ in citrate buffer pH 5.0 were added as substrate. Reactions were terminated with 1 M H_2_SO_4_ and absorbance at 490 nm (OD_490_) was measured. To normalize Mfa1 expression to the amount of intact cells, filtration ELISA was also performed using antibodies raised against *P*. *gingivalis* whole cells [31].

### Auto-aggregation activity of *P*. *gingivalis*

Auto-aggregation activity was measured according to published methods [13, 32]. Cells were harvested by centrifugation at 8,000 ×*g* for 10 min and gently washed twice with, and resuspended in, 20 mM PBS pH 6.0. The OD_600_ of the resulting cell suspension was measured and adjusted to 1.0 by dilution with PBS. Cells were then aliquoted into 13-mm culture tubes at 2 mL per tube, and shaken at 120 strokes/min at room temperature. The OD_660_ of the cell suspension was measured at various time points with a Mini Photo 518R spectrophotometer (Taitec, Saitama, Japan).

### Biofilm assay

Homotypic biofilm formation was quantified using a microtiter plate assay specifically adapted for *P*. *gingivalis* [13, 31]. Briefly, overnight cultures of *P*. *gingivalis* were diluted 1:10 in sTSB. Aliquots of 200 μL were anaerobically incubated for 24 h at 37°C in a flat-bottom 96-well polystyrene plate. To remove planktonic cells, wells were gently washed three times with 10 mM PBS pH 7.4 and air-dried. Remaining cells were then stained for 15 min with 200 μL 0.1% (w/v) crystal violet. Excess dye was removed by washing twice with 10 mM PBS pH 7.4, and then with water. Dye taken up by cells was eluted using 200 μL 95% ethanol and OD_595_ was measured to determine the mass volume of the biofilm.

### Statistics

Data are reported as mean ± standard deviation (SD). One-way analysis of variance and Dunnett multiple-comparison test were used to evaluate differences between groups. Significance was defined as *p* value < 0.01.

## Results

### Processing of Mfa4 and its incorporation into Mfa1 fimbriae

We constructed the *mfa4*-deficient mutant FMFA4 from the *fimA*-deletion strain JI-1 to avoid potential confounding effects from FimA fimbriae. We also constructed FMFA4C, a complemented strain that expresses wild-type *mfa4 in trans*. Immunoblotting with antibodies against Mfa4 [13] showed a clear band with molecular weight 30 kDa in JI-1 and FMFA4C, but not in FMFA4 (Fig 1B). These results indicate that Mfa4 is lost upon disruption of *mfa4*, and is overwhelmingly regained upon complementation.

We examined the subcellular localization of Mfa4 to follow the intracellular processing events that lead to its maturation. As shown in Fig 1C, Mfa4 was detected as a 30-kDa protein in the whole-cell lysate and accumulated predominantly in the envelope fraction. Upon separation of the envelope fraction into inner and outer membranes, Mfa4 was detected mainly in the inner membrane, suggesting that while Mfa4 is a minor fimbrial component, it is predominantly an inner-membrane protein. Nevertheless, some Mfa4 fractionates with the outer membrane, confirming a previous observation that Mfa4 co-purifies with fimbriae recovered from the outer membrane [28].

### Effect of *mfa4* mutation on assembly of Mfa1 fimbriae

**Fig 2:**
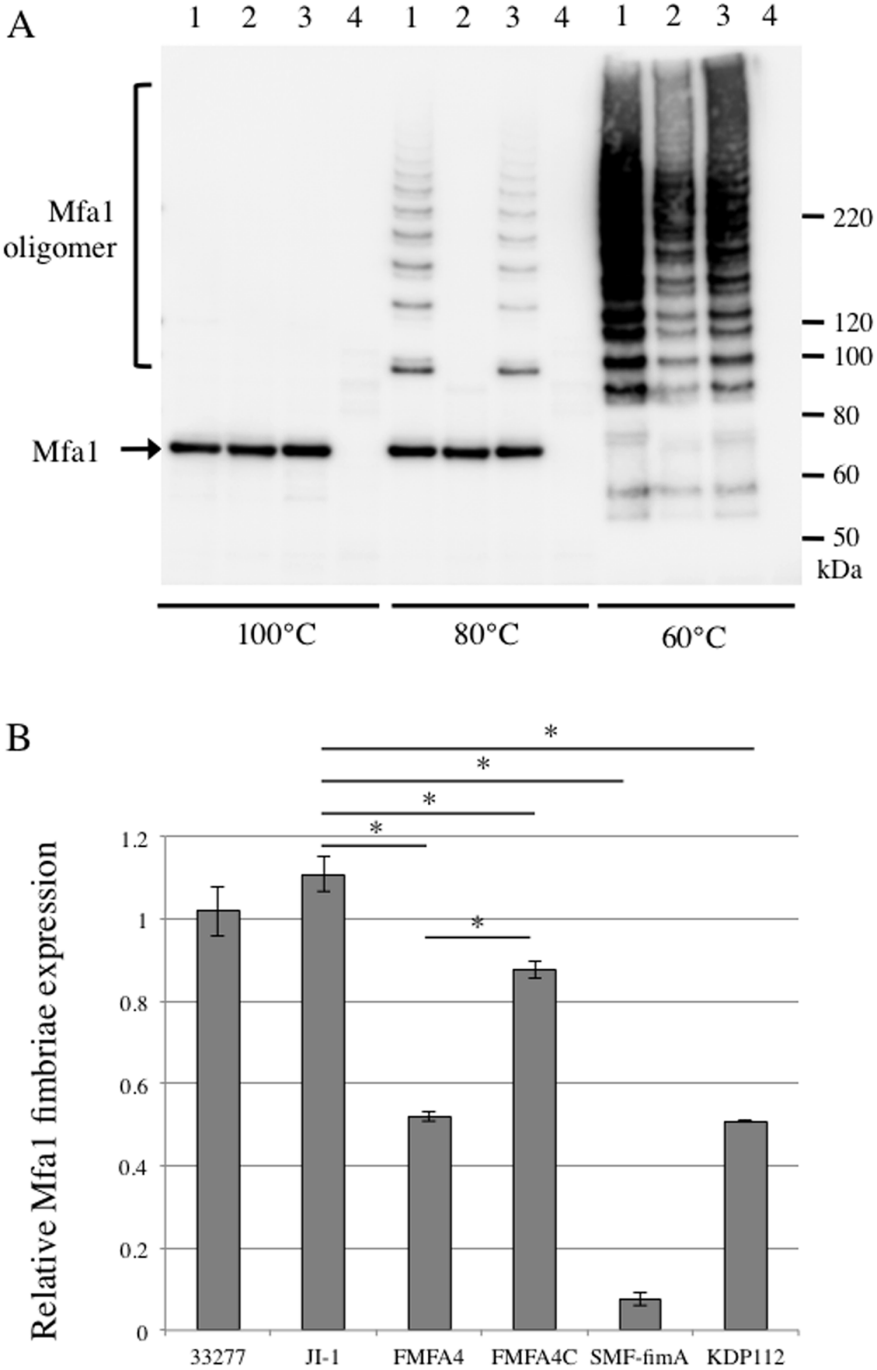
Polymerization and expression of Mfa1. (A) Immunoblot analysis using antiserum against Mfa1 fimbriae. Whole-cell lysates were obtained from *P*. *gingivalis* JI-1 (lane 1), FMFA4 (lane 2), and FMFA4C (lane 3). *mfa1*-deficient SMF-fimA (lane 4) was used as negative control. Samples were denatured at 100°C for 5 min, 80°C for 10 min, or 60°C for 10 min, separated by SDS-PAGE, and analyzed by immunoblotting using antiserum against Mfa1 fimbriae purified from JI-1. Incomplete disassembly of Mfa1 polymers at 60–80°C generates a ladder of oligomers. (B) Filtration ELISA of intact cells. *P*. *gingivalis* ATCC 33277, JI-1, FMFA4, FMFA4C, SMF-fimA, and *rgpA/B*-deficient KDP112 were applied over filters in a filtration plate at 5×10^6^ cells/well. Bacterial cells were probed with antibodies against Mfa1-only fimbriae purified from FMFA5, and then with peroxidase-conjugated goat anti-rabbit IgG at a dilution of 1:1000. Data are mean ± SD from two experiments with triplicates. *, statistically significant difference from JI-1 based on Dunnett’s test (*p* < 0.01).

Mfa1 expression and polymerization were examined by immunoblotting using a specific antiserum against Mfa1 fimbriae purified from JI-1 [13]. No difference was observed in between the band pattern of the blot using anti-Mfa1 fimbriae from JI-1 and FMFA5. Whole-cell lysates from all strains except SMF-fimA, a Mfa1-deficient mutant, contained Mfa1 monomers of comparable molecular weight when completely denatured by heating at 100°C (Fig 2A). In contrast, heating whole-cell lysates from JI-1 and FMFA4C at 80°C generated a ladder, indicating partial dissociation of Mfa1 fimbriae (Fig 2A). We presume that the laddering is due to the effect of molecular shape and incomplete binding of SDS to polymerized Mfa1. However, FMFA4 did not show laddering, except when lysates were heat-denatured at 60°C (Fig 2A). Thus, the two fimbriae differ in temperature-dependent dissociability and it is likely that Mfa1 subunits are held together less strongly in the absence of *mfa4*.

Subsequently, Mfa1 fimbriae expression on the cell surface was determined by ELISA analysis of intact bacterial cells. As shown in Fig 2B, Mfa1 expression on the surface of FMFA4 is 43% of JI-1. Complementation of the mutant with a copy of *mfa4 in trans* significantly increased cell surface display to 79% of JI-1. Nevertheless, expression of Mfa1 fimbriae in FMFA4C is still significantly lower than in J1-1, even though Mfa4 is expressed much more abundantly (Fig 1B). Nevertheless, the data imply that Mfa4 facilitates the display of Mfa1 fimbriae on the cell surface.

### Effect of *mfa4* mutation on other fimbrial proteins

**Fig 3:**
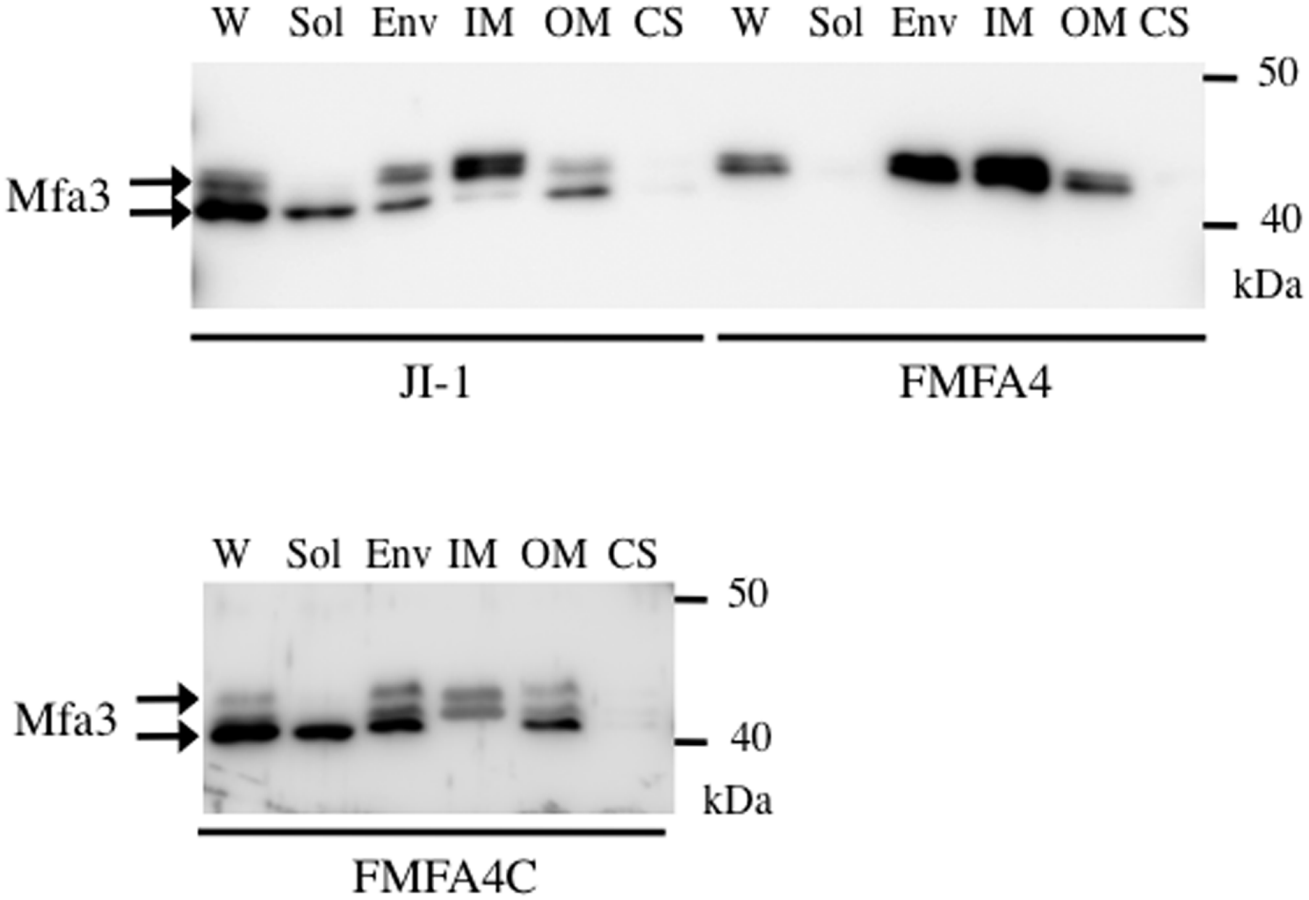
Mfa3 expression and localization in *P*. *gingivalis* JI-1, FMFA4, and FMFA4C. Proteins in the culture supernatant (CS) were concentrated by ammonium sulfate precipitation, while whole-cell lysates (W) were fractionated into soluble (Sol), envelope (Env), inner membrane (IM), and outer membrane (OM) fractions. Samples were separated by SDS-PAGE and probed with Mfa3 antibodies by immunoblotting.

In JI-1 whole-cell lysates, Mfa3 was detected as bands of size 40 and 43 kDa, as shown in Fig 3. Only the 40-kDa band, which corresponds to the mature protein, was detected in the soluble fraction, which also contains Mfa1 fimbriae. The envelope fraction had both forms of Mfa3 in comparable amounts. However, the unprocessed and mature forms were enriched in inner and outer membranes, respectively, indicating that Mfa3 matures as it redistributes from the inner to the outer membrane. Finally, Mfa3 in any form was undetectable in the culture supernatant. In contrast, mature 40-kDa Mfa3 was not detected at all in FMFA4 (Fig 3). Notably, complementation completely rescues processing (Fig 3). These results strongly indicate that Mfa4 is required for maturation of Mfa3 in *P*. *gingivalis*. In contrast, Mfa4 deficiency did not alter the expression and cellular localization of Mfa2, and equivalent amounts of Mfa2 were detected in the outer membrane of FMFA4 and JI-1 (S1 Fig).

**Fig 4:**
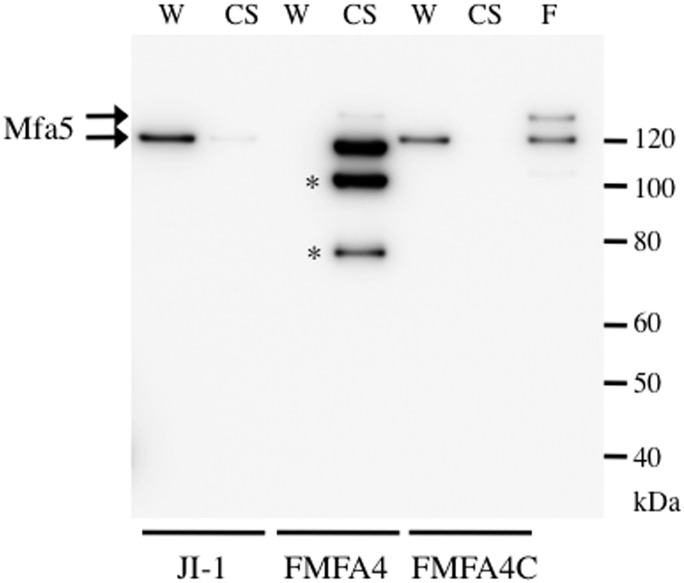
Mfa5 expression and localization in *P*. *gingivalis* JI-1, FMFA4, and FMFA4C. Culture supernatants (CS) and whole-cell lysates (W) from JI-1, FMFA4, and FMFA4C were separated by SDS-PAGE and probed with Mfa5 antibodies by immunoblotting. Structurally intact Mfa1 fimbriae purified from JI-1 (F), which contains Mfa5, were used as positive control. Possible Mfa5 degradation products with molecular weight 100 and 78 kDa are marked with *.

On the other hand, Mfa5 was detected at 130 kDa in whole-cell lysates from JI-1, but not from FMFA4 (Fig 4). In addition, full-length Mfa5, as well as degradation products with molecular weight 100 and 78 kDa, were observed in the culture supernatant from FMFA4. Complementation reverses this leakage, and results in accumulation of Mfa5 in whole-cell lysates of FMFA4C. These results strongly suggest that Mfa4 is required to anchor and stabilize Mfa5 in Mfa1 filament. The 130- and 150-kDa Mfa5 bands were clearly co-purified with Mfa1 fimbriae from J1-1, which is structurally intact (Fig 4, lane F).

### Composition of Mfa1 fimbriae in FMFA4

**Fig 5:**
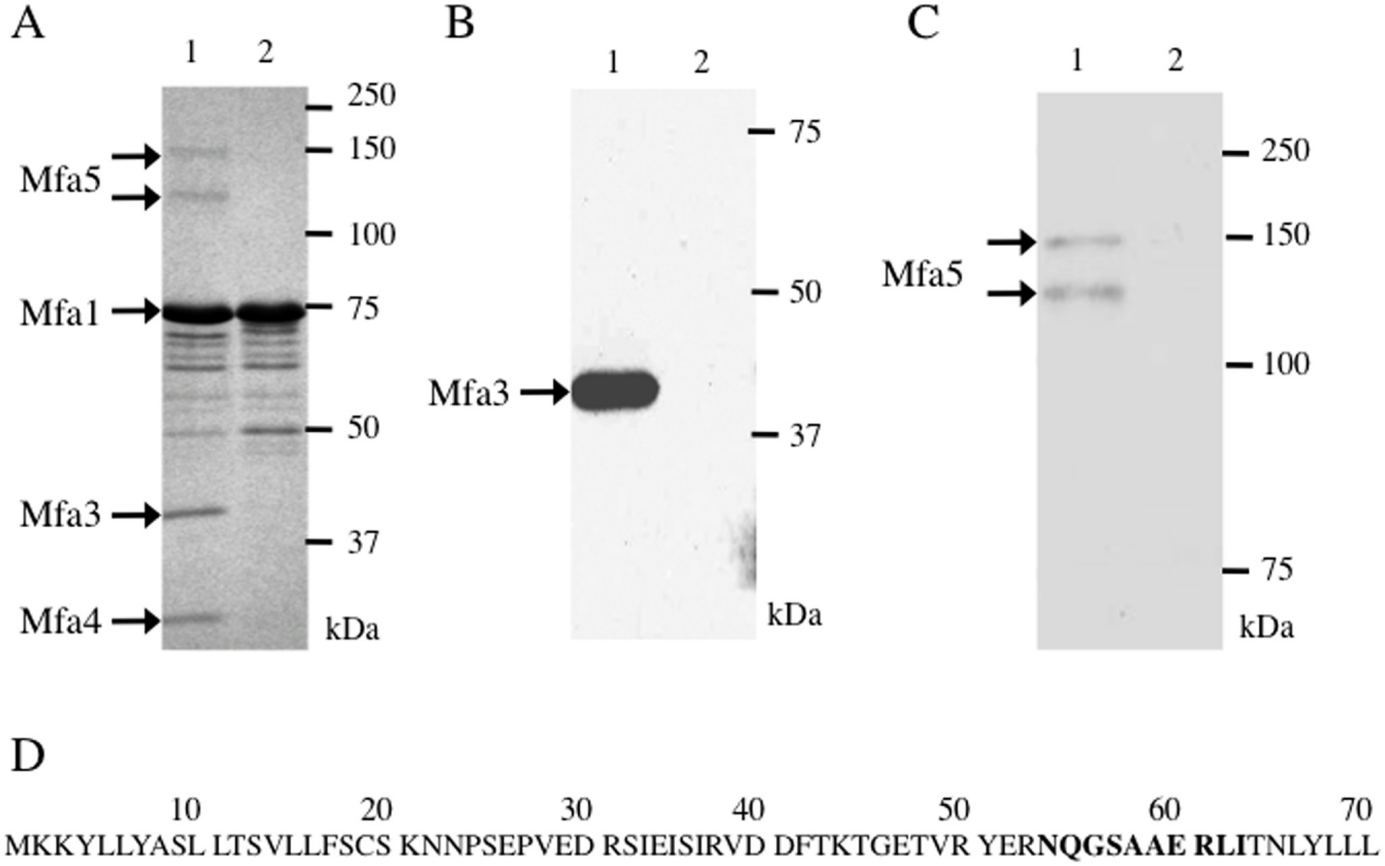
Components of purified Mfa1 fimbriae. (A-C) Mfa1 fimbriae purified from JI-1 (lane 1) and FMFA4 (lane 2) were examined by SDS-PAGE and stained with Coomassie brilliant blue R-250 (A), or analyzed by immunoblotting using anti-Mfa3 (B) and anti-Mfa5 (C). (D) The N-terminus of Mfa4. Amino acids in boldface were identified by Edman degradation of mature Mfa4 incorporated into JI-1 fimbriae.

Mfa1 fimbriae were purified from FMFA4 and JI-1 to examine the effect of *mfa4* deficiency on composition and assembly. As shown in Fig 5A, JI-1 fimbriae (lane 1), contain several proteins, and bands at corresponding molecular weights were identified by mass spectrometry to be Mfa1 and Mfa3-5 [12]. Notably, N-terminal sequencing indicates that Mfa4 incorporated into Mfa1 fimbriae begins at Asn^54^ (Fig 5D).

Mfa1 fimbriae purified from FMFA4 contained a ladder of Mfa1 between 50 and 75 kDa, as observed in JI-1, but did not contain the accessory components Mfa3, Mfa5, and Mfa4 (Fig 5A, lane 2). The absence of accessory components was confirmed by immunoblotting (Fig 5B and 5C).

### Auto-aggregation and biofilm formation in FMFA4

Because *P*. *gingivalis* ATCC 33277 contains a mutation in *fimB* that leads to the production of unusual FimA fimbriae [31], and to avoid possible confounding effects from FimA fimbriae, auto-aggregation and biofilm formation were analyzed in strains from which *fimA* had been deleted.

**Fig 6:**
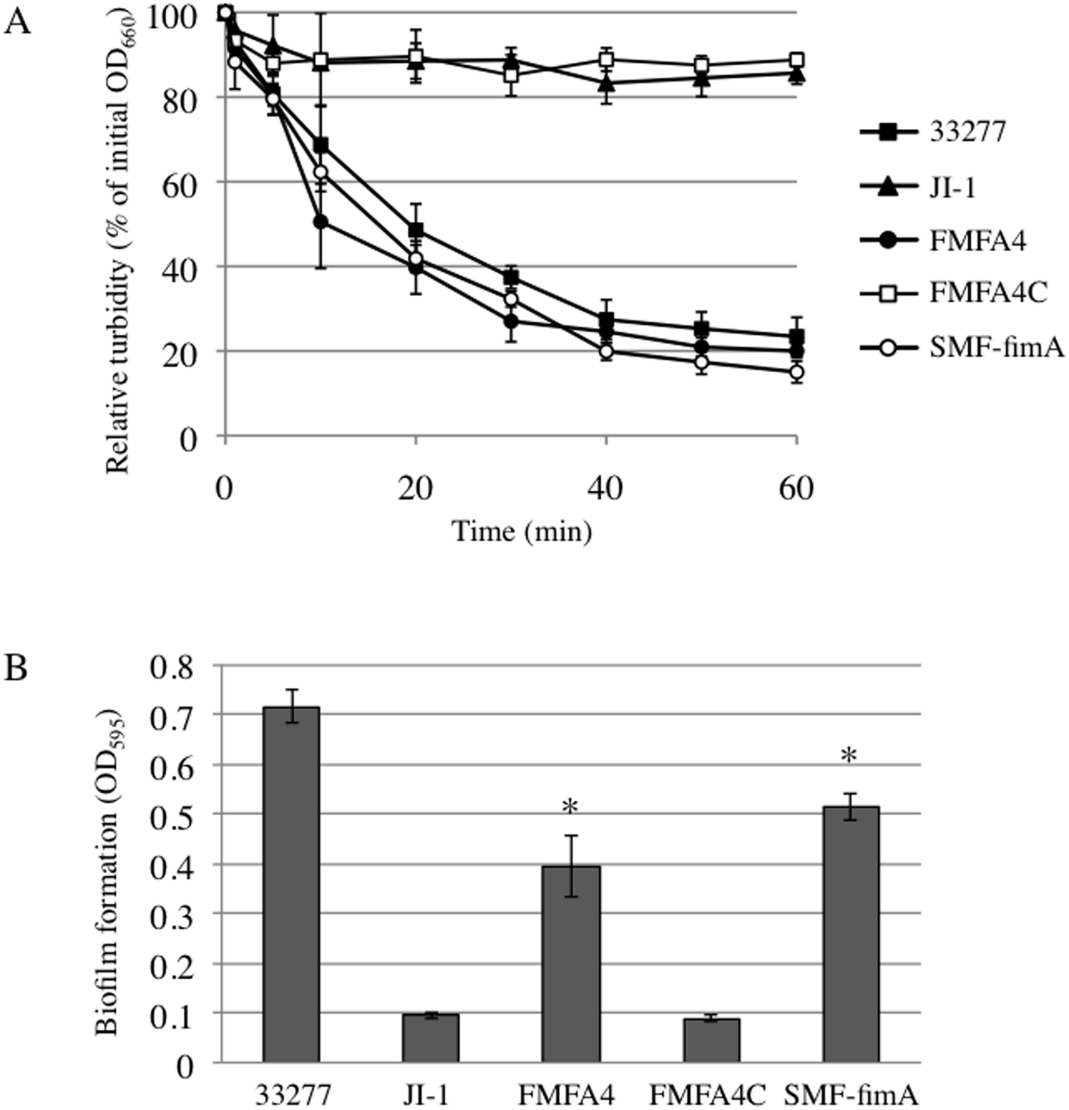
Auto-aggregation and biofilm formation. (A) Relative turbidity, expressed as % of the initial optical density at 660 nm, is plotted as a function of time. Data points are mean ± SD from triplicate experiments. (B) Biofilm formation by ATCC 33277, JI-1, SFM4, SMF4C, and SMF-fimA, as measured by the crystal violet method. Data are means ± SD (n = 8). *, *p* < 0.01 on Dunnett’s test against JI-1.

Wild type *P*. *gingivalis* auto-aggregates in shaking cultures, an event captured by a drastic decline in OD_660_, a measure of turbidity (Fig 6A) [13]. FimA fimbriae are known to accelerate auto-aggregation, whereas Mfa1 fimbriae suppress it [13]. Control experiments confirm that the OD_600_ of liquid cultures of wild type *P*. *gingivalis* declines rapidly over 40–60 min, reaching approximately 20% of the initial value. In contrast, the turbidity of cultures of the *fimA*-deficient strain JI-1 decreased only 10% after 60 minutes (Fig 6A). However, the *mfa1-* and *fimA*-deficient strain SMF-fimA auto-aggregates with kinetics comparable to wild type, also reaching 19% of the initial value after 1 h (Fig 6A). Interestingly, the *mfa4-* and *fimA*-deficient strain FMFA4, which still produces Mfa1 fimbriae, albeit ones without Mfa3 and Mfa5, strongly auto-aggregates like SMF-fimA. Finally, FMA4C auto-aggregates with kinetics similar to JI-1, presumably because complementation of *mfa4* in this strain restores the structure and function of Mfa1 fimbriae.

Crystal violet assays showed that strains SMF-fimA and FMFA4 form biofilms robustly (Fig 6B). However, the complemented strain FMFA4C, which has functional Mfa1 fimbriae, but not FimA fimbriae, has diminished capacity to form biofilms, and resembles JI-1. Indeed, strains that auto-aggregate also form more robust biofilms.

### Expression of the *mfa* gene cluster in *P*. *gingivalis* KDP112

It has been shown that FimA and Mfa1 are processed to maturity by Rgp [18, 19], which specifically cleaves substrates on the carboxyl side of arginine residues. The N-terminal amino acids of Mfa1 and Mfa3 incorporated into Mfa1 fimbriae were reported to be Ala^50^ [16] and Ala^44^ [13], respectively. In this study, we determined the N-terminus of mature Mfa4 to be Asn^54^ (Fig 5D). In all cases, the prior residue was arginine, suggesting that these proteins are also processed by RgpA/B. Therefore, we used KDP112, a double mutant lacking *rgpA/B* [21], to examine whether RgpA/B is involved in processing Mfa1-4.

**Fig 7:**
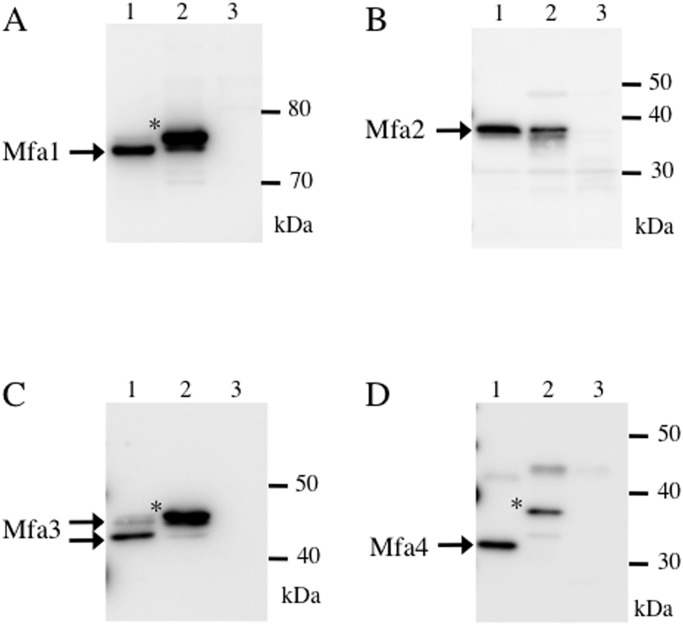
Processing of Mfa1-4 in *P*. *gingivalis* KDP112. Whole-cell lysates from JI-1 (lane 1) and KDP112 (lane 2) were separated by SDS-PAGE and immunoblotted using antibodies against Mfa1 (A), Mfa2 (B), Mfa3 (C), or Mfa4 (D). Lane 3 in each panel is a negative control containing whole-cell lysates from SMF-fimA, JI-12, FMFA3, or FMFA4, respectively. Possible unprocessed, immature forms are marked with *.

Mfa2 in KDP112 is of similar size as in the parental strain (Fig 7B), but the other fimbrial proteins were found to be of higher molecular weight than those in strain JI-1, which expresses Rgp enzymes (Fig 7A, 7C and 7D, lanes 1–2). In KDP112, the molecular weights of these proteins were 78 kDa, 43 kDa, and 38 kDa, respectively. Consistent with defects in the maturation of fimbrial components, quantitative ELISA revealed that KDP112 expressed 50% less Mfa1 fimbriae than wild type (Fig 2B).

## Discussion

Mfa4 is one of the accessory proteins incorporated into Mfa1 fimbriae [12, 13], but its role has remained unclear. In this report, we describe genetic and biochemical approaches to investigate the role of the *mfa4* gene in the assembly, structure, and function of Mfa1 fimbriae.

Incorporation of accessory components into mature fimbriae is common among gram-negative pathogenic bacteria such as uropathogenic *E*. *coli*, *Neisseria meningitidis*, and *Pseudomonas aeruginosa* [33–36]. The roles of some of these components are known. For example, accessory components that cap fimbrial tips facilitate adhesion, as in type I fimbriae and P pili in *E*. *coli* [37, 38]. In *P*. *gingivalis*, a recent study demonstrated that the accessory proteins FimC-E are essential to maintain the structure and adhesive properties of FimA fimbriae [39].

Inactivation of *mfa4* altered the cellular distribution of other accessory proteins. Immature Mfa3 accumulated in the inner membrane, whereas Mfa5 appeared to leak out into the culture supernatant. These effects reduce the amount of fimbriae displayed on the cell surface. The fimbriae themselves have altered composition when *mfa4* is disrupted, contain only polymerized Mfa1, and are without accessory proteins. The control of fimbrial polymerization by CsgB, a minor component of an *E*. *coli* fimbrial structure known as curli, provides precedent. Specifically, CsgB is crucial for efficient polymerization of CsgA, the major subunit in curli [40].

Analysis of cell fractions from the *fimA*-deficient strain JI-1 shows that Mfa4 accumulates in the inner membrane. Because immature Mfa3 also accumulates in this fraction when *mfa4* is absent, as in the strain FMFA4, it is tempting to speculate that Mfa4 regulates the transport of immature Mfa3, perhaps via a mechanism that resembles the chaperone-usher pathway, which is typically required to assemble various adhesive pili. In this system, individual pilus subunits reach the periplasm via the general secretory pathway, and are then folded by specific periplasmic chaperones and delivered to the outer membrane. At the outer membrane, subunits are assembled into pili by an usher [41]. However, a periplasmic chaperone-usher system for fimbrial proteins has not been found so far in *P*. *gingivalis*. Nevertheless, it has been shown that FimA and Mfa1 are trafficked via a unique transport and assembly mechanism that generates a lipoprotein intermediate. A leader peptide is also cleaved off by signal peptidase II and RgpA/B prior to polymerization at the cell surface [16, 42].

Mfa5 is thought to belong to a family of C-terminal domain proteins secreted via the Por secretion system [43], a unique secretion apparatus in *P*. *gingivalis* [44, 45]. Thus, Mfa5 may be secreted to the outer membrane via mechanisms not used for other proteins in the *mfa* gene cluster. Notably, C-terminal domain proteins are extensively modified with A-LPS and migrate as diffuse bands in SDS-PAGE with molecular mass generally 20 kDa higher than would be expected from the sequence [46, 47]. In this study, Mfa5 incorporated into Mfa1 fimbriae was found to migrate as two bands with molecular weight 130 kDa and 150 kDa (Fig 4, lane F). As the first corresponds to the calculated molecular mass of 134495.35 Da, the second therefore appears to be Mfa5 decorated with A-LPS. Because modified Mfa5 was not detected in cellular fractions of FMFA4, and was released to the culture media (Fig 4), it is likely that A-LPS anchors Mfa5 to the extracellular side of the outer membrane. However, the direct role of Mfa4 in these processes is still unclear.

FMFA4, which expresses defective Mfa1 fimbriae lacking Mfa3-5, strongly auto-aggregates and robustly forms biofilms compared to JI-1. Notably, these activities are similar to levels in SMF-fimA, even though Mfa1 fimbriae are still expressed on the cell surface at 43% of JI-1 (Fig 2B). On the other hand, auto-aggregation and biofilm formation are diminished to the same extent as JI-1 in the complemented strain FMFA4C, which expresses structurally intact Mfa1. These data demonstrate that accessory proteins strongly influence fimbrial structure and function.

Similarly, the adhesive properties of *P*. *gingivalis* FimA fimbriae depend crucially on the accessory proteins FimC-E [32, 48]. In addition, Wang et al. [39] proposed that these accessory proteins are essential for virulence in experimentally induced periodontitis. A very recent study also found FimC-D to be involved in invasion of human oral keratinocytes and gingival fibroblasts [49]. It is obvious from these reports that FimA accessory proteins are functionally significant. Therefore, further work may also find critical roles for Mfa1 accessory proteins, perhaps in co-aggregation with oral streptococci or in interaction with host cells.

In the strain KDP112, from which there is complete loss of Rgp protease activity, unprocessed and immature Mfa1, Mfa3, and Mfa4 were detected. The defect in fimbrial protein processing decreases the expression of surface Mfa1 fimbriae by 42.5% (Fig 2B). Note that unprocessed Mfa1 could be detected at the cell surface by filtration ELISA because the antiserum against Mfa1-only fimbriae reacted with both Mfa1 monomers and oligomers. However, electron microscopy of KDP112 indicates that fimbriae are hardly observed on the cell surface [19], suggesting that RgpA/B is important for the formation of structurally sound fimbriae.

In conclusion, Mfa4 facilitates the incorporation of accessory proteins into Mfa1 fimbriae, not only by promoting maturation of Mfa3 but also by stabilizing Mfa5 at the cell surface. In this manner, Mfa4 mediates the formation of Mfa1 fimbriae and ultimately modulates biofilm formation. Furthermore, Rgp proteases process Mfa1, Mfa3, and Mfa4 to maturity.

## Supporting Information

S1 Fig Expression and subcellular distribution of Mfa2. Cultures of *P*. *gingivalis* JI-1 and FMFA4 were harvested by centrifugation and fractionated into whole-cell lysate (W), soluble (Sol), envelope (Env), inner membrane (IM), and outer membrane (OM) fractions. The culture supernatant (CS) was also analyzed after ammonium sulfate precipitation. Samples were boiled at 100°C for 5 min in a buffer containing SDS and 2-mercaptoethanol, and were then subjected to western blot using antibodies raised against Mfa2.
(TIFF)
