# Supplementary figures and images for: Mfa4, an Accessory Protein of Mfa1 Fimbriae, Modulates Fimbrial Biogenesis, Cell Auto-Aggregation, and Biofilm Formation in Porphyromonas gingivalis
Source: PLoS One. 2015 Oct 5;10(10):e0139454. doi: 10.1371/journal.pone.0139454 (PMC4593637; doi:10.1371/journal.pone.0139454)

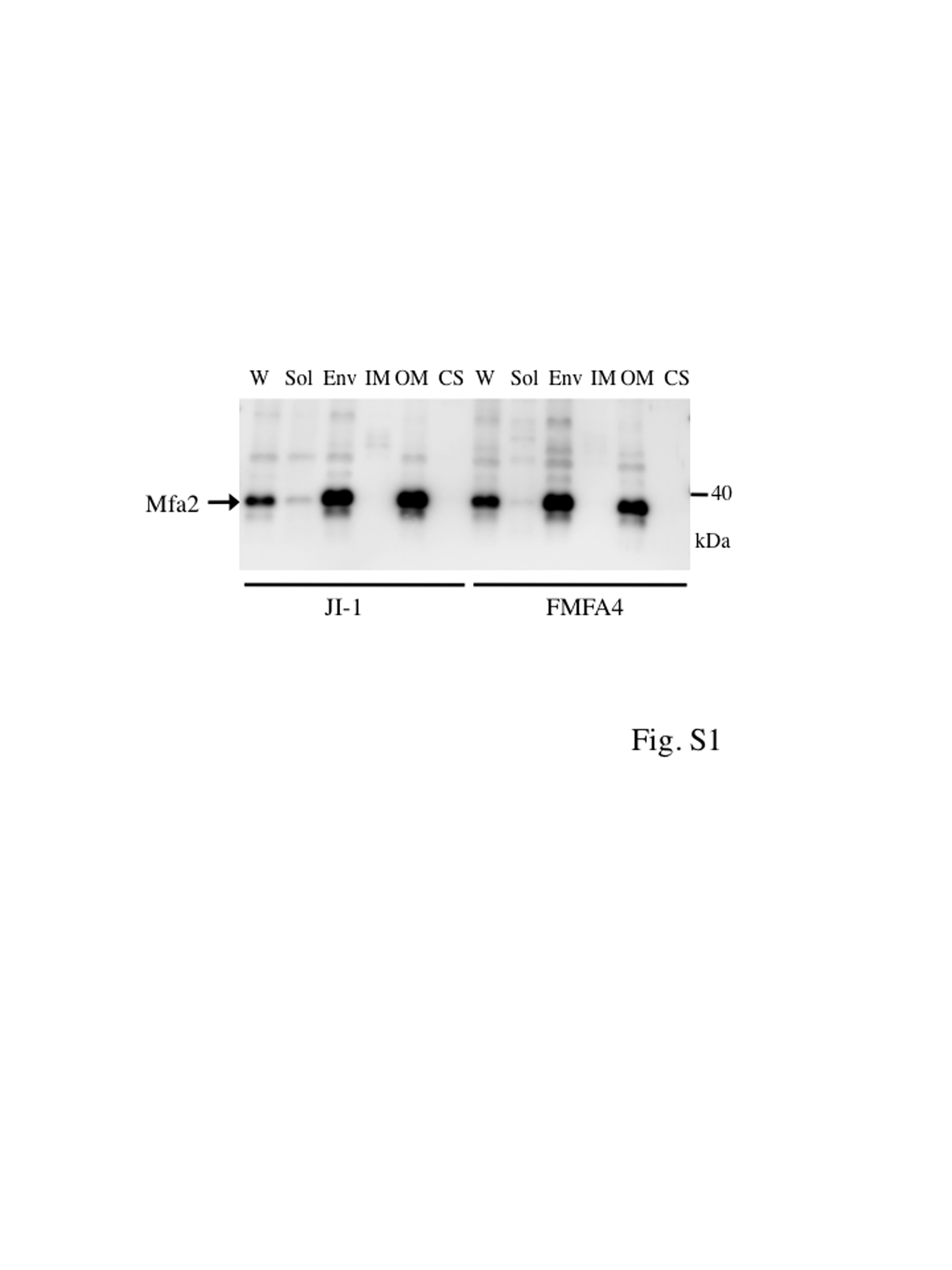

Supplement: S1 Fig — Cultures of P. gingivalis JI-1 and FMFA4 were harvested by centrifugation and fractionated into whole-cell lysate (W), soluble (Sol), envelope (Env), inner membrane (IM), and outer membrane (OM) fractions. The culture supernatant (CS) was also analyzed after ammonium sulfate precipitation. Samples were boiled at 100°C for 5 min in a buffer containing SDS and 2-mercaptoethanol, and were then subjected to western blot using antibodies raised against Mfa2. (TIFF) [file pone.0139454.s001.tiff]
